# Nested PCR and the TaqMan SNP Genotyping Assay enhanced the sensitivity of drug resistance testing of *Mycobacterium leprae* using clinical specimens of leprosy patients

**DOI:** 10.1371/journal.pntd.0007946

**Published:** 2019-12-27

**Authors:** Xiaohua Chen, Jun He, Jian Liu, Yuangang You, Lianchao Yuan, Yan Wen

**Affiliations:** 1 Beijing Tropical Medicine Research Institute, Beijing Friendship Hospital, Capital Medical University, Beijing, China; 2 Beijing Key Laboratory for Research on Prevention and Treatment of Tropical Diseases, Capital Medical University, Beijing, China; 3 The Center for Disease Control and Prevention of Yunnan Province, Kunming, China; Beijing Institute of Microbiology and Epidemiology, CHINA

## Abstract

**Background:**

Although leprosy is efficiently treated by multidrug therapy, resistance to first-line (dapsone, rifampin) and second-line (fluoroquinolones) drugs has been described worldwide. However, the characteristics of drug resistance in Southwest China remain unknown. Furthermore, the sensitivity of polymerase chain reaction (PCR)/sequencing for resistance detection is limited, especially for paucibacillary (PB) leprosy patients. The current study aimed to develop a nested PCR/sequencing and TaqMan SNP Genotyping Assay to increase the sensitivity of the method used to detect drug resistance in *Mycobacterium leprae* and to reveal the nature of *M*. *leprae* drug resistance in Southwest China.

**Methodology/Principal findings:**

Seventy-six specimens, including skin biopsy (n = 64), formalin-fixed paraffin-embedded (FFPE) (n = 11) and skin-slit smear (SSS) (n = 1) samples from multibacillary (MB, n = 70) and PB (n = 6) leprosy patients from Southwest China, were included in this study. The presence of mutations in drug resistance-determining regions (DRDRs) of the rpoB, folP1, and gyrA genes, which are associated with rifampicin, dapsone, and quinolone resistance, respectively, was detected by PCR/sequencing, as recommended by the WHO, and the nested PCR and TaqMan SNP Genotyping Assay developed in this study. Mutations in the folP gene were detected in 19 (25.00%) samples, indicating dapsone-resistant *M*. *leprae*, with one (1.31%) sample showing mutations in two genes, folP and gyrA, reflecting multidrug-resistant strains to dapsone and ofloxacin. However, no rpoB mutation was detected. Compared with PCR/sequencing, nested PCR increased the sensitivity of detecting rpoB (from 51.39% to 78.94% for leprosy patients and from 0.00% to 50.00% for PB), gyrA (from 75.00% to 80.26% for leprosy patients and from 50.00% to 66.67% for PB), and folP1 (from 5.26% to 84.21% for leprosy patients and from 0.00% to 66.67% for PB). Moreover, the TaqMan SNP Genotyping Assay showed greater sensitivity for folP1 detection (from 5.26% to 78.94–86.84% for leprosy patients and from 0.00% to 33.33%-83.33% for PB patients) than the PCR/sequencing method. In addition, the latter method was able to more easily distinguish heterozygous genotypes and mutant homozygous genotypes from homozygous genotypes.

**Conclusions/Significance:**

Nested PCR/sequencing and the TaqMan SNP Genotyping Assay are rapid and highly sensitive methods for detecting drug resistance in leprosy cases. The current study revealed that diamino-diphenylsulfone (DDS; also known as dapsone) resistance in *M*. *leprae*, as indicated by folP1 gene detection, is still the most concerning form of drug resistance in leprosy patients from Southwest China.

## Author summary

Despite being a curable disease, leprosy remains a public health problem in more than 100 countries, where over 200,000 new leprosy cases are reported each year. The incidence rate has remained steady since 2005, indicating continued active transmission of the disease. Since the 1940s, the strategy for leprosy control has involved diamino-diphenylsulfone (DDS) monotherapy and then multidrug therapy (MDT), as recommended by the World Health Organization in 1982. After 30 years of DDS monotherapy, drug resistance has been described worldwide, and after 30 years of MDT, drug resistance has unsurprisingly been observed. However, the nature of drug resistance in Southwest China is still unknown. As the sensitivity of the PCR/sequencing method is limited, especially among paucibacillary (PB) patients, we developed a nested PCR/sequencing and TaqMan SNP Genotyping Assay that dramatically increased the sensitivity of detecting drug resistance among drug resistance-determining regions (DRDRs). According to results, the folP1 mutant is predominant, but rpoB mutants were not found. The results of this study indicate the preliminary characteristics of drug resistance in the DRDRs of leprosy patients from Southwest China.

## Introduction

Leprosy is a chronic human disease caused by the yet-uncultured pathogen *Mycobacterium leprae* [1]. Although curable with multidrug therapy (MDT), leprosy remains a public health problem in South America, Africa, South and Southeast Asia, and Micronesia, where over 200,000 new leprosy cases are reported each year [1]. The incidence rate has remained steady since 2005 [2], and global statistics of childhood cases show that transmission of leprosy continues to some extent in more than 100 countries [3]. Therefore, surveillance is needed to ensure that chemotherapy of leprosy remains effective for the foreseeable future [4].

*M*. *leprae* is susceptible to a wide range of antibiotics; diamino-diphenylsulfone (DDS; also known as dapsone) monotherapy was used to treat leprosy from 1940 to 1981, and multidrug therapy (MDT) was then implemented worldwide based on recommendations by the WHO (1982–2000). The components of MDT, comprising rifampicin, dapsone, and clofazimine, and several second-line drugs, ofloxacin, minocycline, and clarithromycin, are sometimes employed as therapeutic agents [1]. Resistance to antileprosy drugs in *M*. *leprae* has been observed in several leprosy-endemic regions by the Global Sentinel Surveillance for Drug Resistance in Leprosy program coordinated by the WHO [5]. The emergence of drug-resistant (DR) and multidrug-resistant (MDR) *M*. *leprae* has also been increasingly reported in other areas [2, 6–12].

The presence of mutations in drug resistance-determining regions (DRDRs) of the rpoB, folP1, and gyrA genes is associated with rifampicin, dapsone, and quinolone resistance, respectively. Testing methods include *M*. *leprae* DRDR primers and a polymerase chain reaction (PCR) sequencing method recommended by the WHO [13], GenoType LepraeDR [14], and qPCR-high-resolution melt analysis [15], among others. However, the use of nested PCR and the TaqMan SNP Genotyping Assay for this type of testing has not been reported. Nested PCR is necessary for studies on certain human tissue microbiota because it can amplify the target DNA with concentrations several-fold lower than those of standard PCR [16]. As new SNP mutations associated with antibiotic resistance have been detected by *M*. *leprae* whole-genome sequencing [1], the TaqMan SNP Genotyping Assay is a potential molecular method for detecting drug resistance in *M*. *leprae*.

Leprosy is still endemic in 61 counties in the Yunnan, Sichuan, and Guizhou Provinces of China (prevalence ≥ 1/100,000). Indeed, more than half of the country’s new cases (336/634) in 2017 were detected in these three provinces, making it the area with the highest leprosy burden in China. In the present study, skin biopsy, formalin-fixed, paraffin-embedded (FFPE), and skin-slit smear (SSS) samples of new and relapsed cases from multibacillary (MB) and paucibacillary (PB) patients with leprosy were used. Our aim was to develop an easily accessed and highly sensitive nested polymerase chain reaction (nested PCR) and Custom TaqMan SNP Genotyping Assay that easily detects drug resistance in leprosy.

## Methods

### Ethics statement

This study was approved by the Medical Ethics Committee of Beijing Friendship Hospital, Capital Medical University, Beijing, P. R. China. Written informed consent was obtained from all study participants or from their parents or guardians. All of the procedures in the study, including biological sample collection and testing involving human participants, were performed in accordance with the ethical standards of the institutional and/or national research committee and with the 1964 Helsinki Declaration and its later amendments or comparable ethical standards.

### Human specimens

This molecular epidemiology study was performed with Chinese patients with leprosy from 2003 to 2011. Seventy-six patients were included, mainly from Yunnan Province, including the Center for Disease Control and Prevention (CDC) of Wenshan Zhuang and Miao Autonomous Prefecture (WS), Honghe Hani and Yi Autonomous Prefecture (HH), and Kunming (KM) (the capital of Yunnan) and Guizhou, Sichuan, Hunan, and Shandong Provinces; Tianjin; and Tibet. In this study, all patients diagnosed with leprosy by clinicians using defined criteria, SSSs and biopsies were appropriately treated with DDS and/or MDT according to WHO guidelines. Seventy-six clinical specimens were obtained from the patients with leprosy for routine diagnosis of leprosy in China. These specimens were divided into two groups according to disease status: new cases (n = 50) and relapsed cases (n = 26). The specimens were divided by type into skin biopsy (n = 37), FFPE (n = 38) and SSS (n = 1) groups. The specimens were also divided based on the bacterial index (BI) value into high BI (BI≥2; n = 57) and low BI (BI<2; n = 16) groups. BI information was not available for three patients.

### DNA extraction and quality control

Genomic DNA was extracted from FFPE and skin biopsy specimens using the Qiagen DNeasy^™^ Blood and Tissue Kit (Qiagen, Beijing, China) according to the manufacturer’s instructions. Briefly, paraffin was removed from the FFPE specimens by vortexing and 10 min of incubation with 1.2 mL xylene, followed by two washes with pure (200 proof) ethanol. The FFPE and skin biopsy specimens were then incubated with 20 μL proteinase K and 180 μL ATL lysis buffer overnight in a heat block at 56°C. The lysed emulsion was further purified with DNeasy Spin-Column Kit. The DNA was eluted in 100 μl of AE elution buffer provided in the kit; 1 to 2 μl of DNA was typically sufficient for one PCR.

For DNA yield, the total amount of DNA for clinical samples was assessed using spectrophotometry (Malcom e-spect, Tokyo, Japan) and a Qubit 4.0 fluorometer with Qubit dsDNA HS Assay Kit (Life Technologies, Massachusetts, Waltham, USA) according to the manufacturer’s protocols. The total amount of DNA was obtained in ng/μl.

### Simple PCR, nested PCR, and sequencing

The primers used for simple PCR were recommended by the WHO (see Table 1). The 20-μl reaction mixture contained 1 μl of primer mix (250 nM of each primer for folP1, rpoB, and gyrA), 10 μl of 2X PCR Mixture (Cat No: MFKIT02 for folP1, MFKIT03 for rpoB, and MFKIT04 for gyrA, Beijing Jinsheng Lida Technology Trade Co., Ltd., Beijing, China), and 2–50 ng of DNA template (1 μl). Reactions were carried out using a Peltier thermal cycler (BIO-RAD, California, USA), and the thermal cycling conditions were as follows: 2 min at 95 °C, followed by 35 cycles of 15 sec at 95 °C, 15 sec at 58 °C and 60 sec at 72 °C, and a final extension step of 7 min at 72 °C.

**Table 1:** Primers for simple PCR/sequencing and nested PCR/sequencing for drug susceptibility testing in *M*. *leprae* used in this study.

Target gene	Primers	Nucleotide sequences (5'-3')	Product length	PCR type
folP1	Forward	5'-CTGACAATTCGTTCTCAGATGG-3'	394 bp*	Nested PCR (outer)
	Reverse	5'-TAATTCGGAGCCTCATA-3'		
	Forward	5'-CTTGATCCTGACGATGCT G-3'	255 bp	Simple PCR/Nested PCR (inner) /Sequencing
	Reverse	5'-CCCACCAGATCGTTGACG-3'		
rpoB	Forward	5'-GTCGGTATGTCGCGGATGGA-3'	581 bp	Nested PCR (outer)
	Reverse	5'-CGTGGCGAGACATCCATGTAAT-3'		
	Forward	5'-GTCGGTATGTCGCGGATGGA-3'	279 bp	Simple PCR/Nested PCR (inner) /Sequencing
	Reverse	5'-CGACAATGAACCGATCAGACC-3'		
gyrA	Forward	5'-TATACAGCGGGTTGAGCGG-3'	358 bp	Nested PCR (outer)
	Reverse	5'-GATGGTCTCAAACCGGTACATC-3'		
	Forward	5'-ACAATAACGCATCGCTGCCG-3'	225 bp	Simple PCR/Nested PCR (inner) /Sequencing
	Reverse	5'-ACCCGGCGAACCGAAATT G-3'		

*Bp = base pair; PCR = polymerase chain reaction; nested PCR = single-tube nested PCR.

Simple PCR/STNPCR (inner) primers of folP1, rpoB, and gyrA recommended by the WHO;

STNPCR (outer) primers designed by authors through Primer Express 5.0.

The simple PCR primers recommended by the WHO were used as the inner primers for the nested PCR, and the outer primers of the nested PCR primer were designed using Primer Express 5.0 (Table 1). The first round of nested PCR was performed as following. The 20-μl reaction mixture for nested PCR contained 10 μl of 2× PCR Mixture, 1 μl of outer primer mix (250 nM), Cat No: MFKIT02 for folP1, MFKIT03 for rpoB, and MFKIT04 for gyrA (Beijing Jinsheng Lida Technology Trade Co., Ltd., Beijing, China) and 2–50 ng of DNA template (1 μl). The thermal cycling conditions were as follows: 7 min at 94 °C, followed by 40 cycles of 30 sec at 94 °C, 30 sec at 58 °C and 60 sec at 72 °C, and a final extension step of 7 min at 72 °C. Products were visualized on a 1.5% agarose gel containing GeneFinder (Zeesan Biotech, Xiamen, Fujian, China). The second round of nested PCR was performed as the procedure of simple PCR.

The nucleotide sequences of both strands of the relevant segment of the folP1, rpoB, and gyrA genes were determined by direct sequencing of the PCR product using an ABI 3730 Automated DNA Sequencer (Sangon Biotech Co., Ltd). The primers used for sequencing are listed in Table 1. Sequence data were analyzed using the nucleotide database in Basic Local Alignment Search Tool (BLAST) (http://blast.ncbi.nlm.nih.gov) to identify mutations associated with drug resistance.

### TaqMan SNP genotyping

Genotyping of folP1 drug resistance loci ACC53GCC, CCC55CTC, CCC55CGC, and ACC53ATC was performed using TaqMan SNP Genotyping Master Mix (Applied Biosystems, Foster City, CA, USA) and the ABI 7500 fast real-time polymerase chain reaction (PCR) system (Applied Biosystems). The final reaction volume for PCR was 10 μl, which contained 1 μl of 10 ng/μl genomic DNA or 1^st^ PCR products, 5 μl of TaqMan Universal PCR Master Mix, 0.25 μl of 200 nM VIC/FAM-labeled probe, and 3.75 μl double-distilled water. The probes were designed by Beacon Designer 8.00, synthesized as Custom TaqMan SNP Genotyping Assays, Nonhuman (Applied Biosystems). The probe sequences are indicated in Table 2. PCR amplification was carried out in 96-well plates containing unknown genotype samples, wildtype samples (e.g., 55CCC), heterozygous samples (e.g., 55CCC/CGC), and homozygous samples (e.g., 55CGC) as positive controls and no-template controls. Thermal cycle conditions were as follows: 1 cycle of 60 °C for 1 min, 95 °C for 10 min, 40 cycles of 95 °C for 15 sec and 60 °C for 1 min, and 1 cycle of 60 °C for 1 min. Reactions were analyzed using Allelic Discrimination Sequence Detection Software from Applied Biosystems.

**Table 2:** Primers and probes of Custom TaqMan SNP genotyping assays for the folP1 loci for drug susceptibility testing in *M*. *leprae*.

folP1 ACC53GCC	Forward Primer	GGCGCGGCGATTGTC
	Reverse Primer	ACTCGAGGATCGGTCCTAATGG
	Probe 1 Wild type [VIC]	CCGGGTCGATTCG
	Probe 2 Mutant [FAM]	CGGGCCGATTCG
folP1 CCC55CTC	Forward Primer	CGTCGGTGGCGAATCGA
	Reverse Primer	TCAACTCGAGGATCGGTCCTAA
	Probe 1 Wild type [VIC]	TGGCACCGGGCCGG
	Probe 2 Mutant [FAM]	TGGCACCGAGCCGG
folP1 CCC55CGC	Forward Primer	ACGTCGGTGGCGAATCG
	Reverse Primer	CAACTCGAGGATCGGTCCTAATG
	Probe 1 Wild type [VIC]	ACCCGGCCCGGTGC
	Probe 2 Mutant [FAM]	CCGGCGCGGTGC
folP1 ACC53ATC	Forward Primer	GGCGCGGCGATTGTC
	Reverse Primer	ACTCGAGGATCGGTCCTAATGG
	Probe 1 Wild type [VIC]	CGAATCGACCCGGCCC
	Probe 2 Mutant [FAM]	CGAATCGATCCGGCCC

bp = base pair; PCR = polymerase chain reaction; STNPCR = single-tube nested PCR.

### Statistical analysis

The DNA concentration from different sample types was compared using GraphPad Prism software version 5.0 (GraphPad Software Inc., San Diego, CA, USA). Following Tukey’s honest significant difference (HSD) test was used for analysis of the average DNA quantification values obtained from skin biopsy or FFPE samples by spectrophotometry and Qubit dsDNA HS Assay. Comparisons of the three molecular assays for drug resistance were performed by Statistical Package for the Social Sciences (SPSS) version 16.0. Concordance between the results was determined by kappa values (κ), and p values were calculated. Significant differences between assays were determined by McNemar’s test, and p values were calculated.

## Results

### Basic characteristics of leprosy patients

Seventy-six leprosy cases from different regions were included. The basic information for each study group is summarized in Table 3.

**Table 3:** Clinical characteristics of the leprosy patients enrolled in this study.

Variables		Cases (%)
WHO Classification	MB*	70 (92.10%)
	PB*	6 (7.89%)
Ridley-Jopling Classification	LL**	26 (34.21%)
	BL**	34 (44.73%)
	BB**	4 (5.26%)
	BT**	9 (11.84%)
	TT**	3 (3.95%)
Sex	Female	17 (22.37%)
	Male	59 (77.63%)
Age (years)	≤20	7 (9.21%)
	21–39	30 (39.47%)
	40–59	28 (36.84%)
	≥60	11 (14.47%)
Province	Yunnan	54 (71.05%)
	Guizhou	7 (9.21%)
	Sichuan	5 (6.57%)
	Hunan	4 (5.26%)
	Hubei	1 (1.32%)
	Fujian	1 (1.32%)
	Shandong	1 (1.32%)
	Tianjin	1 (1.32%)
	Jiangsu	1 (1.32%)
	Tibet	1 (1.32%)
Disease Status	New	50 (65.79%)
	Relapsed	26 (34.21%)
Specimen Type	Skin biopsy	37 (48.68%)
	FFPE	38 (50.00%)
	SSS	1 (1.32%)
BI	≥2	58 (76.32%)
	<2	18 (23.68%)

*MB: multibacillary, PB: paucibacillary.

**TT: tuberculoid tuberculoid, BT: borderline tuberculoid, BB: mid-borderline, BL: borderline lepromatous, LL: lepromatous lepromatous

### Comparison of DNA yield between skin biopsy and FFPE samples

**Fig 1:**
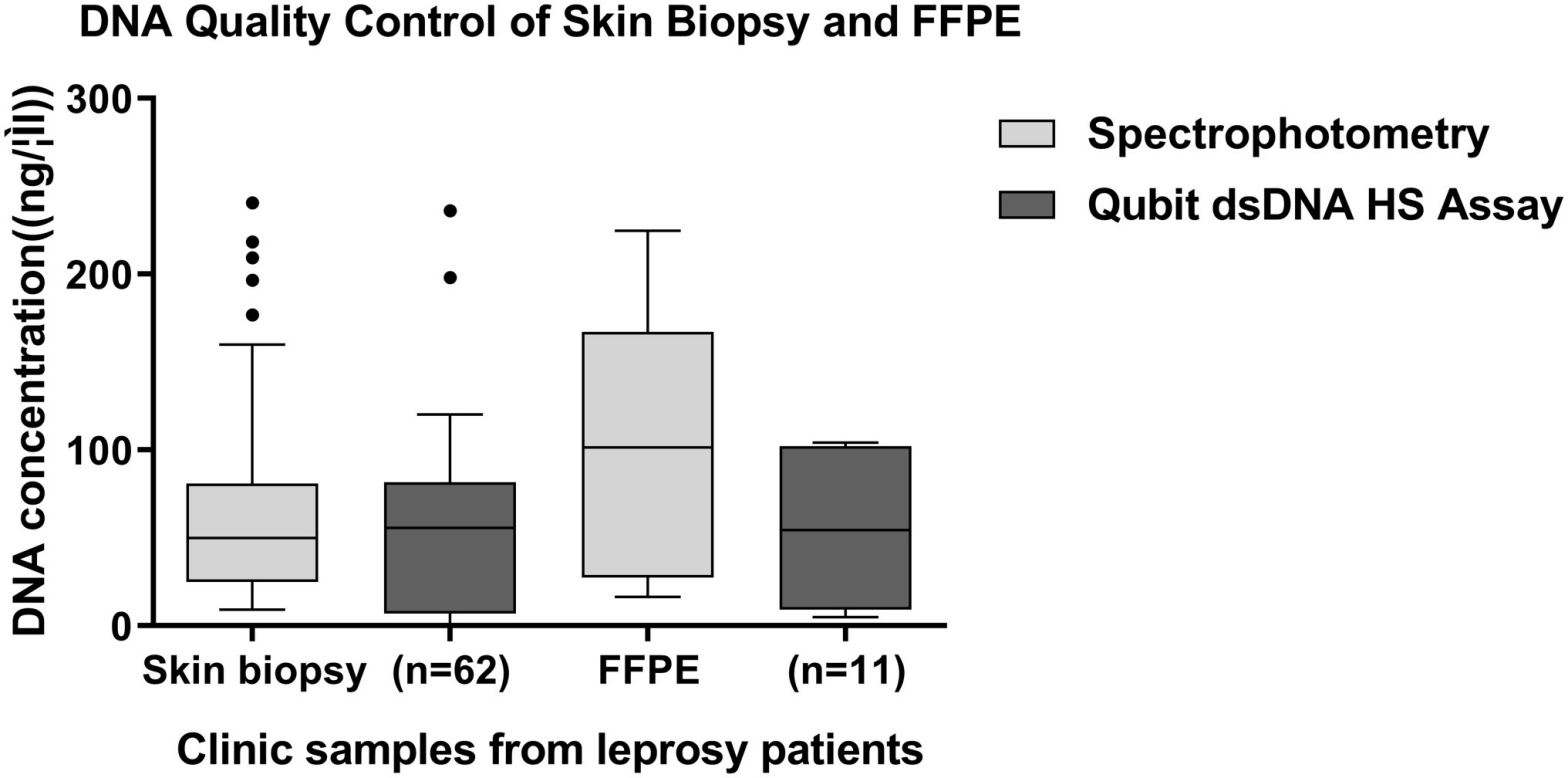
DNA concentrations (ng/μl) resulting from *M*. *leprae* clinic specimens.

The DNA concentrations from different sample types were compared by spectrophotometry and Qubit dsDNA HS Assay. FFPE samples generated a slightly higher DNA yield than did skin biopsy samples, but this difference was not significant (Fig 1, S2 Table). The DNA concentration of skin biopsy samples measured by the Qubit dsDNA HS Assay method (average = 53.01 ng/μl, SD = 46.53) was slightly lower than that obtained by spectrophotometry (average = 65.24 ng/μl, SD = 55.42). In contrast, the DNA concentration of FFPE samples measured by the Qubit dsDNA HS Assay method (average = 107.9 ng/μl, SD = 72.99) was considerably lower than that obtained by spectrophotometry (average = 58.73 ng/μl, SD = 42.54) (Fig 1, S2 Table).

### Analytical specificity of simple PCR, nested PCR and TaqMan methods

The specificity of the primers/probes was tested by carrying out amplification with purified genomic DNA from fifteen different mycobacterial species and four nonmycobacterial species. Regarding the gyrA and folP1 genes, all primer/probe sets produced amplicons when DNA isolated from *M*. *lepraemurium* was used as the template, but no amplicons were observed when DNA from other species of *Mycobacterium* or nonmycobacterial species was used (see Table 4). For rpoB, *M*. *bovis* BCG-Pasteur, *M*. *bovis* [AFZ/ZZ/97], *M*. *bovis* [Ravenel], *M*. *kansasii*, and *M*. *ulcerans* amplicons were present, as verified by BLAST (https://blast.ncbi.nlm.nih.gov/Blast.cgi), and the sequencing results were consistent with *Mycobacterium* species detected.

**Table 4:** Specificity of nested PCR, and TaqMan SNP genotyping assay for drug resistance testing of *Mycobacterium leprae*.

Species	rpoB		gyrA		folP1		
	Simple PCR	Nested PCR	Simple PCR	Nested PCR	Simple PCR	Nested PCR	TaqMan SNP Genotyping
*Mycobacterial species*	from CSU* and NHDP**						
*M*. *lepraemurium*^#^	+	+	+	+	+	+	+
*M*. *avium*	-	-	-	-	-	-	-
*M*. *bovis BCG-Pasteur*#	-	+	-	-	-	-	-
*M*. *bovis [AFZ/ZZ/97]*#	-	+	-	-	-	-	-
*M*. *bovis [Ravenel]*#	-	+	-	-	-	-	-
*M*. *flavescens*	-	-	-	-	-	-	-
*M*. *intracellulare*	-	-	-	-	-	-	-
*M*. *kansasii*	-	+	-	-	-	-	-
*M*. *marinum*	-	-	-	-	-	-	-
*M*. *phlei*	-	-	-	-	-	-	-
*M*. *smegmatis*	-	-	-	-	-	-	-
*M*. *simiae*	-	-	-	-	-	-	-
*M*. *ulcerans*	-	+	-	-	-	-	-
*Nonmycobacterial species*	from NHDP**						
*Streptococcus pyogenes*	-	-	-	-	-	-	-
*Clostridium perfringens*	-	-	-	-	-	-	-
*Escherichia coli*	-	-	-	-	-	-	-
*Staphylococcus epidermidis*	-	-	-	-	-	-	-

*CSU = Colorado State University, Fort Collins, Colorado

**NHDP = National Hansen’s Disease Program

^#^: The results of PCR/sequencing and nested PCR/sequencing were consistent with the *Mycobacterium* species detected.

### Performance of drug resistance testing of clinical samples

PCR to amplify folP1, gyrA and rpoB DRDRs was performed on all samples. PCR amplicons corresponding to DRDRs in the folP1, gyrA and rpoB genes were investigated by direct sequencing of the PCR amplicons obtained using a modification of the WHO guidelines for the Global Surveillance of Drug Resistance in Leprosy [13]. Positive PCR results and informative sequences were obtained for folP1 in 15/76 (19.73%) and 3/76 (5.26%) cases, for rpoB in 44/76 (57.89%) and 39/76 (51.35%) cases and for gyrA in 61/76 (80.26%) and 57/76 (75.00%) cases, respectively.

Due to initial PCR inhibition, we developed a nested PCR of DRDRs for the three genes. The positive ratio of nested PCR and sequences increased to 66/76 (86.84%) and 64/76 (84.21%) for folP1, 63/76 (82.89%) and 60/76 (78.94%) for rpoB, and 65/76 (85.52%) and 61/76 (80.26%) for gyrA. We developed a TaqMan SNP assay for the four DRDR loci in the folP1 gene. Because the amplification efficiency of genomic DNA was too low to perform the TaqMan SNP assay, we used the first cycle of the PCR product of nested PCR as the template. The rate for the TaqMan SNP Assay positivity of the folP1 gene was 60/76 (78.94%) for CCC55CTC, CCC55CGC, and ACC53ATC loci and 66/76 (86.84%) for the ACC53GCC locus (Table 5).

**Table 5:** Results of DNA sequencing and TaqMan SNP genotyping in the folP1, rpoB and gyrA genes of *Mycobacterium leprae* in DRDRs.

Specimens		RpoB, n (%)				GyrA, n (%)				folP1, n (%)				folP1, TaqMan**, n (%)			
Type	n	PCR	Seq*	nested PCR	Seq	PCR	Seq	nested PCR	Seq	PCR	Seq	nested PCR	Seq	CCC55 CTC	CCC55 CGC	ACC53 ATC	ACC53 GCC
Total	76	44	39	63	60	61	57	65	61	15	4	66	64	60	60	60	66
		57.89%	51.35%	82.89%	78.94%	80.26%	75.00%	85.52%	80.26%	19.73%	5.26%	86.84%	84.21%	78.94%	78.94%	78.94%	86.84%
New	48	27	24	40	39	35	33	38	36	10	1	40	39	36	37	39	41
		56.25%	50.00%	83.33%	81.25%	72.91%	68.75%	79.16%	75.00%	20.83%	2.08%	83.33%	81.25%	75.00%	77.08%	81.25%	85.41%
Relapsed	28	17	15	24	21	26	24	26	25	5	3	24	23	24	23	21	25
		60.71%	53.57%	85.71%	75.00%	92.85%	85.71%	92.85%	89.28%	17.85%	10.71%	85.71%	82.14%	85.71%	82.14%	75.00%	89.28%
Biopsy	64	35	32	54	51	50	48	53	51	12	4	56	55	51	50	52	56
		54.68%	50.00%	84.37%	79.68%	78.12%	75.00%	82.81%	79.68%	18.75%	6%	87.50%	85.93%	79.68%	78.12%	81.25%	87.50%
FFPE	11	8	6	8	8	10	8	11	9	3	0	9	9	8	9	7	9
		72.72%	54.54%	72.72%	72.72%	90.90%	72.72%	100.00%	81.81%	27.27%	0.00%	81.81%	81.81%	72.72%	81.81%	63.63%	81.81%
SSS	1	1	1	1	1	1	1	1	1	0	0	1	1	1	1	1	1
		100%	100%	100%	100%	100%	100%	100%	100%	0%	0%	100%	100%	100%	100%	100%	100%
BI (≥2)	58	37	34	48	47	47	44	48	47	15	4	51	49	47	48	46	51
		63.79%	58.62%	66.74%	81.03%	81.03%	75.86%	82.75%	81.03%	25.86%	6.89%	87.93%	84.48%	81.03%	87.25%	79.31%	87.93%
BI (<2)	18	6	5	9	13	14	13	16	15	0	0	15	15	13	12	14	15
		33.33%	27.78%	50.00%	72.22%	77.78%	72.22%	88.89%	83.33%	0.00%	0.00%	83.33%	83.33%	72.22%	66.67%	77.78%	83.33%
MB	70	44	39	60	57	57	54	61	57	15	4	62	60	55	58	56	62
		62.85%	51.42%	85.71%	81.42%	81.42%	77.14%	87.14%	81.42%	21.42%	5.71%	88.57%	85.71%	78.57%	82.85%	80.00%	88.57%
PB	6	0	0	3	3	4	3	4	4	0	0	4	4	5	2	4	4
		0.00%	0.00%	50.00%	50.00%	66.67%	50.00%	66.67%	66.67%	0.00%	0.00%	66.67%	66.67%	83.33%	33.33%	66.67%	66.67%

*Seq: sequencing;

**TaqMan: TaqMan SNP genotyping.

### Comparison of the three molecular assays for drug resistance based on kappa and McNemar’s tests

The kappa test was used to analyze the concordance of the results collected from the three molecular assays. Concordance was observed between PCR/sequencing and nested PCR/sequencing of rpoB and gyrA and between nested PCR/sequencing and TaqMan SNP Assay of folP1 (ACC53GCC), with index values of 0.439, 0.849 and 0.257, respectively (p values of 0.000, 0.000, and 0.024, respectively) (Table 6). This finding indicates that the two tests showed high consistency for the detection of drug resistance in leprosy patients.

**Table 6:** Comparison of the results of three molecular assays of drug resistance using clinical samples of leprosy patients.

Methods	Gene (loci)	Kappa test		McNemar’s test
		value	P	P
PCR/Seq *vs* nested PCR/Seq	rpoB	0.439	0.000*	0.000*
	gyrA	0.849	0.000*	0.125
	folP1	0.021	0.374	0.000*
PCR/Seq *vs* TaqMan SNP Assay	folP1 (ACC53GCC)	0.029	0.289	0.000*
	folP1 (ACC53ATC)	0.029	0.289	0.000*
	folP1 (CCC55CGC)	0.029	0.289	0.000*
	folP1 (CCC55CGC)	0.021	0.374	0.000*
Nested PCR/Seq *vs* TaqMan SNP Assay	folP1 (ACC53GCC)	0.257	0.024*	0.791
	folP1 (ACC53ATC)	0.128	0.205	0.503
	folP1 (CCC55CGC)	0.041	0.715	0.523
	folP1 (CCC55CTC)	-0.049	0.667	0.541

*p<0.05, significant difference.

McNemar’s test showed that the sensitivity of nested PCR/sequencing of rpoB and folP1 was significantly positively correlated with the results of PCR/sequencing of rpoB and folP1 (p < 0.05). McNemar’s test also showed that the sensitivity of the TaqMan SNP assay was significantly positively correlated with the PCR/sequencing results, suggesting that the nested PCR/seq and TaqMan SNP assays were helpful for detecting drug resistance in *M*. *leprae* (Table 6).

In addition, we compared the results regarding mutations in specimens detected by the TaqMan SNP Assay and by nested PCR/sequencing, and the two results were identical.

### Characteristics of patients with mutated strains

Data on the mutations observed in the folP1, gyrA, and rpoB genes are summarized in Table 7. Mutations were found in folP1 and gyrA in nineteen patients.

**Table 7:** folP, gyrA, and rpoB gene mutations related to drug resistance in isolates from leprosy patients in China.

Case no.						Mutation (position) of folP1					gyrA
	Age	Sex	Province	Treatment	Status	ACC53GCC	ACC53ATC	CGG54GGG	CCC55CGC	CCC55CTC	
f1-3	69	M	Hunan	MDT	New					CCC55CTC	
f1-4	55	M	Hunan	MDT	New					CCC55CTC	
F1-5	31	F	Yunnan	MDT	New					CCC55CTC	
f1-6	22	F	Yunnan	MDT	New	ACC53GCC					
f1-7	60	M	Yunnan	MDT	Relapsed					CCC55CTC	
f1-8	32	F	Yunnan	MDT	New	ACC53[G/A]CC				CCC55C[T/C]C	
f1-9	55	M	Yunnan	MDT	New				CCC55C[G/C]C		
f1-10	32	M	Yunnan	MDT	New					CCC55C[T/C]C	
F1-12	45	M	Yunnan	MDT	New					CCC55CTC	
f1-21	40	M	Yunnan	DDS/MDT	Relapsed	ACC53GCC					
f-1-2	33	M	Jiangsu	MDT	Relapsed	ACC53GCC					
f-1-10	50	M	Yunnan	MDT	New				CCC55CGC		
f-1-21	50	M	Sichuan	DDS/MDT	Relapsed				CCC55CGC		
f-1-22	43	M	Sichuan	MDT	New				CCC55C[G/C]C		
F-1-24	68	M	Sichuan	MDT	Relapsed					CCC55CTC	
f-1-26	32	M	Yunnan	MDT	New				CCC55CGC		GCA91GTA
f = 1–1	38	F	Yunnan	MDT	Relapsed				CCC55C[G/C]C		
f = 1–9	34	M	Yunnan	MDT	Relapsed		ACC53A [C/T][C/T]	CGG54 [C/G]GG	CCC55C[G/C]C		
f = 1–27	37	M	Yunnan	MDT	New		ACC53ATC				

**Fig 2:**
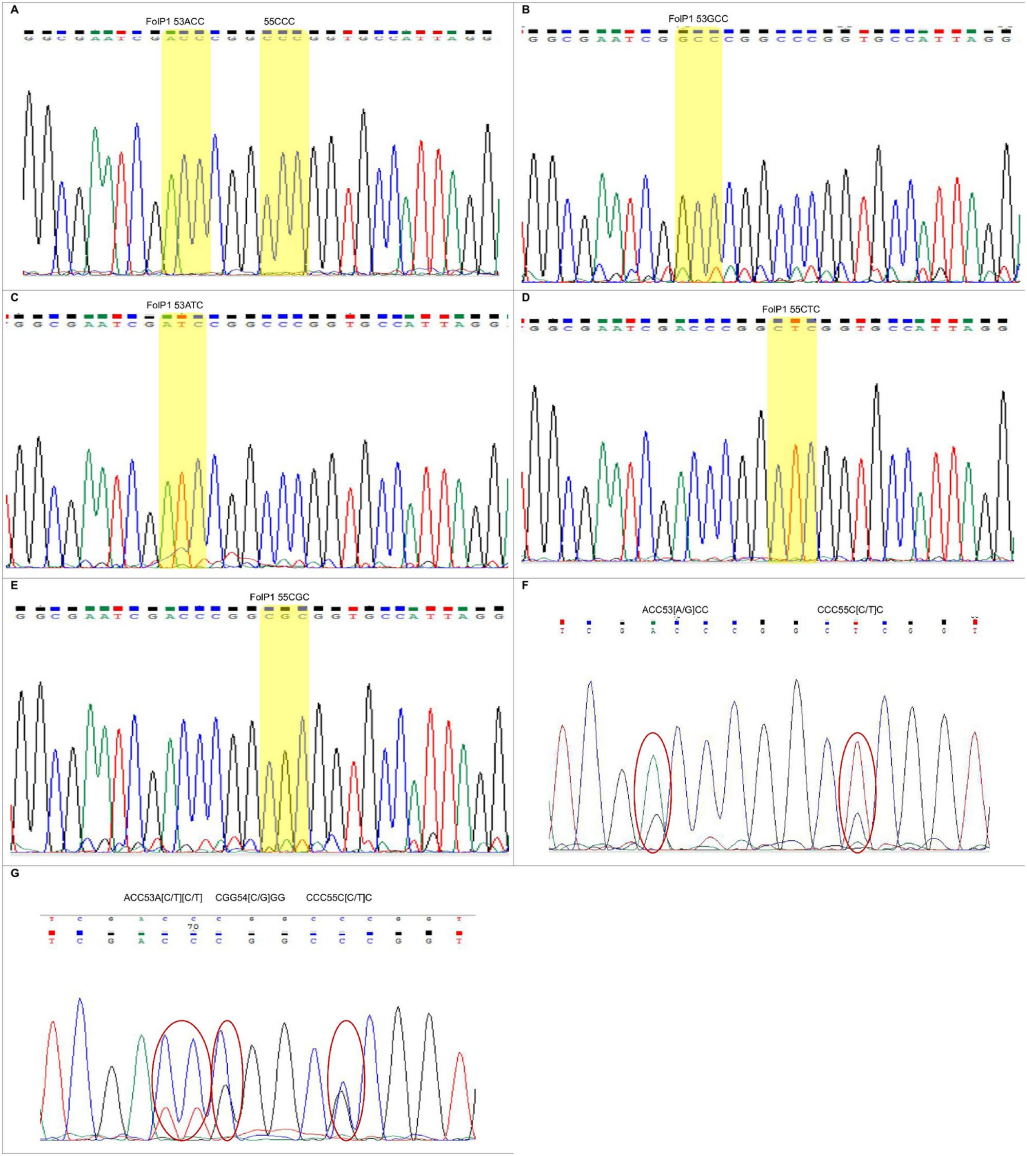
Sanger sequencing chromatograms indicated drug resistance loci in the folP1 Gene of *M*. *leprae*. (A) 53ACC and 55CCC; (B) 53GCC; (C) 53ATC; (D) 55CTC; (E) 55CGC; (F) 53[A/G]CC and 55C[C/T]C; and [G] ACC53A[C/T][C/T], CGG54[C/G]GG and CCC55C[C/G]C in the folP1 gene. (A) Wildtype homozygous; (B-E) mutant homozygous; (F-G) mutant heterozygous.

**Fig 3:**
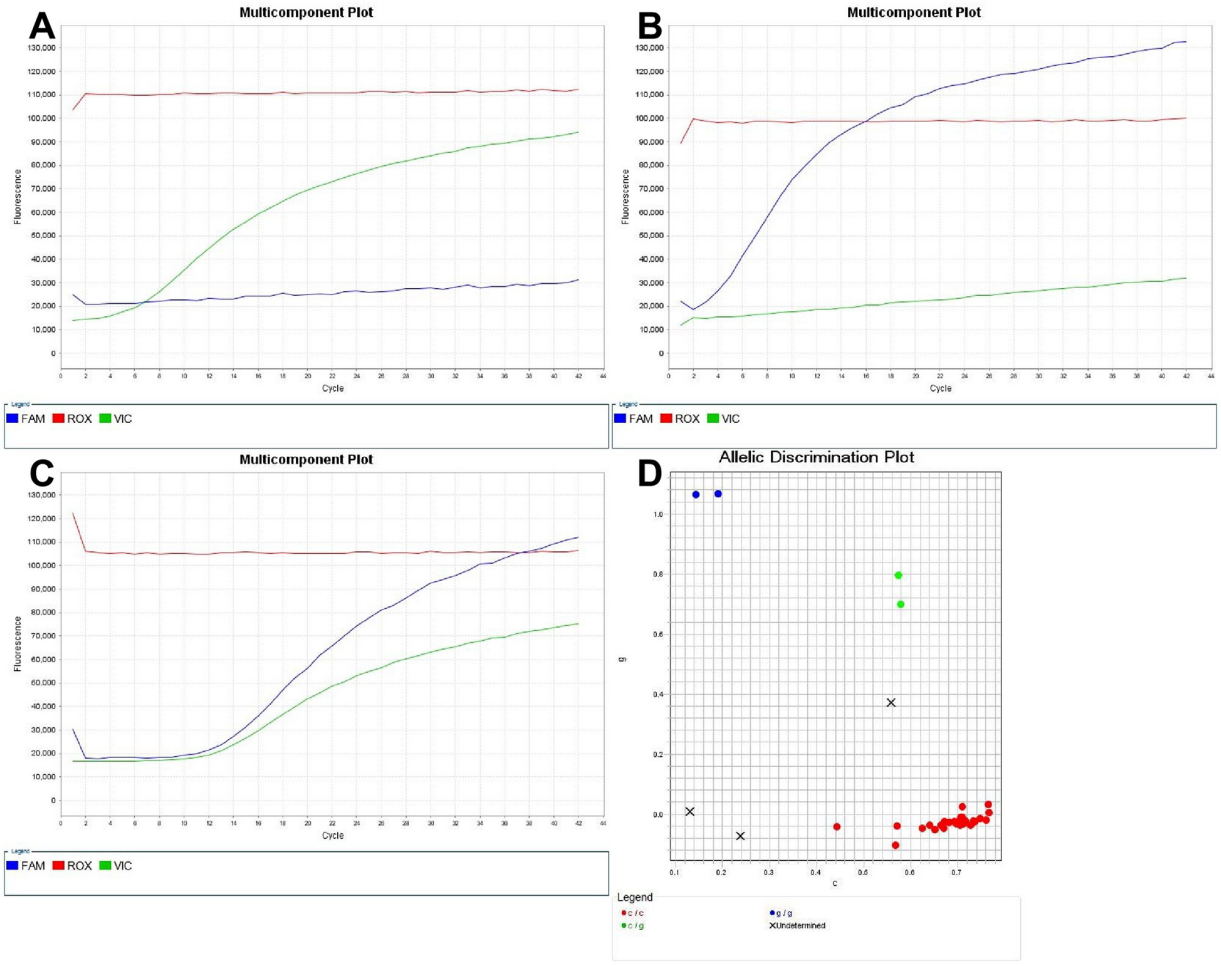
Multicomponent plot figures indicated drug resistance loci (CCC55CGC) in the folP1 Gene of *M*. *leprae*. The DNA samples were genotyped using the Custom TaqMan SNP Genotyping Assay System. Major (also wildtype) alleles were detected by 2'-chloro-7'-phenyl-1,4-dichloro-6-carboxyfluorescein (VIC)-labeled probes (green) and minor alleles by 6-carboxyfluorescein (FAM)-labeled probes (blue). (A) Mutant homozygous G/G. (B) Mutant heterozygous C/G. (C) Wildtype homozygous C/C; (D) Allele discrimination plot showing alleles as wildtype homozygous C/C (lower right cluster), mutant heterozygous C/G (middle cluster), and mutant homozygous G/G (upper left) for the CCC55CGC loci of folP1 gene by Custom TaqMan SNP Genotyping Assay using 7500 Software v2.3, Applied Biosystem, USA. SNP indicates a single-nucleotide polymorphism.

**Fig 4:**
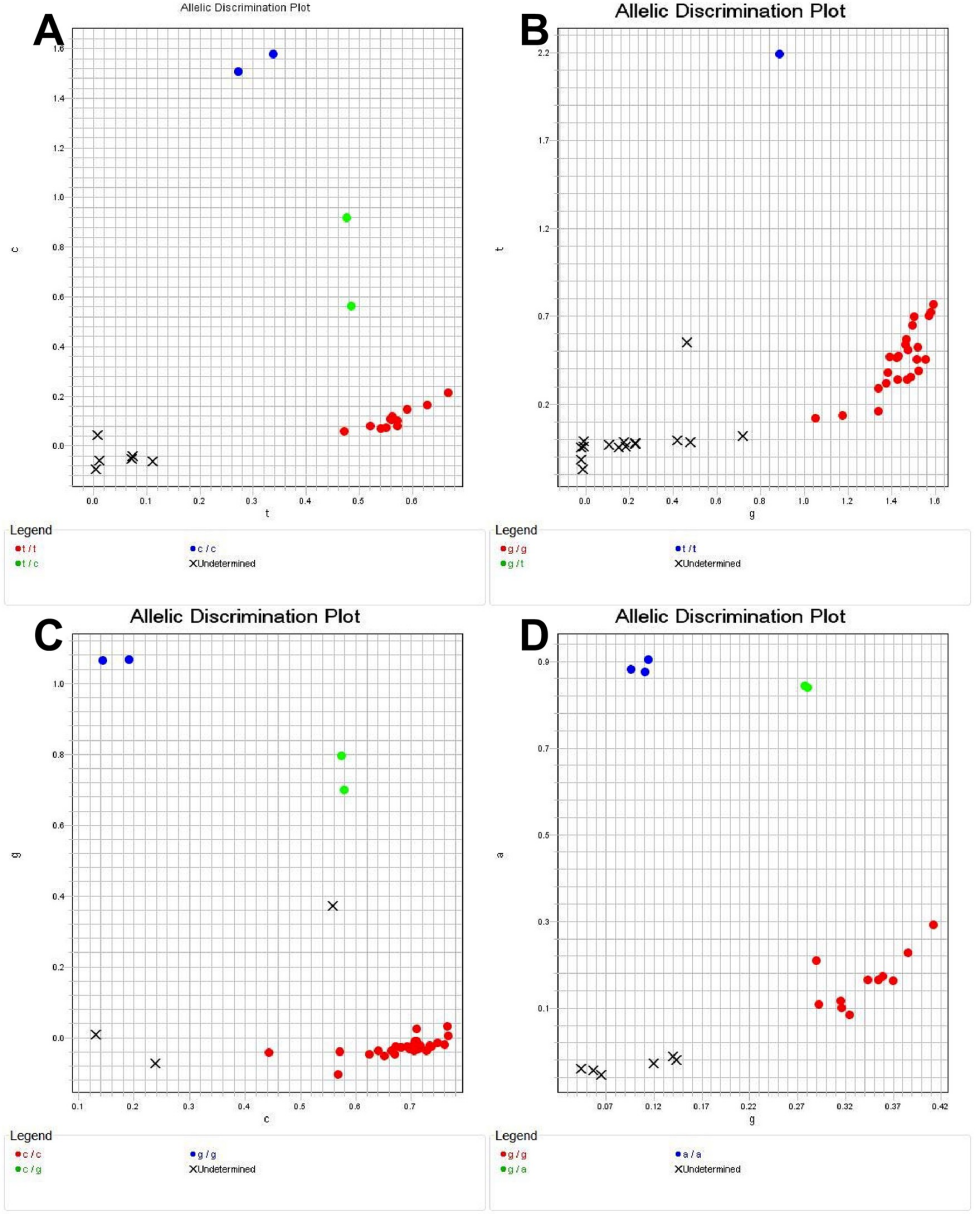
An allele discrimination plot indicated drug resistance loci in the folP1 Gene of *M*. *leprae*. (A) ACC53GCC, (B) ACC53ATC, (C) CCC55CGC, and (D) CCC55CTC. Red, green, and blue dots represent homozygous genotypes, heterozygous genotypes and mutant homozygous genotypes, respectively, and the cross on the bottom left of the plot indicates the no-template control or failed PCR.

Regarding the detection of dapsone resistance, nineteen isolates showed mutations in the folP1 gene, and these mutations were classified into five patterns. Four isolates showed an A to G mutation resulting in a Thr to Ala at amino acid 53 (ACC-GCC). The ratio of mutant homozygous to mutant heterozygous genotypes was 2 to 2. One isolate showed a C to T mutation resulting in a Thr to Ile at amino acid 53 (ACC-ATC) without a mutant heterozygous genotype. One isolate showed a C to T mutation resulting in a Thr to Ile at amino acid 53 (ACC-A[C/T][C/T]) with a mutant heterozygous genotype. Seven isolates showed a C to G mutation resulting in a Thr to Ile at amino acid 55 (CCC-CGC). The ratio of mutant homozygous to mutant heterozygous genotypes was 4 to 3. Eight isolates showed a C to T mutation resulting in a Thr to Ile at amino acid 55 (CCC-CTC). The ratio of mutant homozygous to mutant heterozygous genotypes was 6 to 2. In addition, we found a C to G mutation resulting in a Arg to Gly at amino acid 54 (CGG-[C/G]GG), which was reported previously by Nakata et al. [17] (Table 7). The chromatogram for the drug susceptibility and resistance loci in the folP1 gene of *M*. *leprae* detected by Sanger sequencing is shown in Fig 2. The drug susceptibility and resistance loci in the folP1 gene of *M*. *leprae* detected by the Custom TaqMan SNP assay are shown in Figs 3 and 4.

Regarding ofloxacin resistance, one isolate showed mutations in the GyrA gene of *M*. *leprae*. The isolates showed a C to T mutation resulting in a Ala to Val at amino acid 91 (GCA-GTA) (Table 7).

No isolates showed mutations in *M*. *leprae* rpoB gene DRDRs. One isolate exhibited multidrug resistance and carried mutations in the folP1 and gyrA genes, resulting in resistance to dapsone and ofloxacin.

The mutation ratio of new and relapsed leprosy patients is shown in Table 7. The rates of mutations detected in folP1, gyrA, folP1/gyrA and rpoB were 25.00% (19/76), 1.31% (1/76), 1.31% (1/76), and 0.00% (0/76), respectively.

The drug resistance mutations of *M*. *leprae* in new and relapsed patients with leprosy are shown in Table 8.

**Table 8:** The mutation rates of new and relapsed leprosy patients.

Mutation	Total		New		Relapsed	
	Cases	%	Cases	%	Cases	%
No mutation	57	75.00%	38	76.00%	19	73.08%
Dapsone (folP1)	19	25.00%	12	24.00%	7	26.92%
Quinolones (gyrA)	1	1.31%	1	2.00%	0	0.00%
Dapsone and quinolones (folP1 and gyrA)	1	1.31%	1	2.00%	0	0.00%
RFP (rpoB)	0	0.00%	0	0.00%	0	0.00%
Total	76	100.00%	50	100.00%	26	100.00%

## Discussion

In this study, we investigated drug-resistance mutations in new and relapsed cases of leprosy in Southwest China and developed two molecular biological methods, involving nested PCR/sequencing and the TaqMan SNP Genotyping Assay, for MB and PB leprosy patients.

Regarding specificity, this study assessed whether the simple PCR/sequencing, nested PCR/sequencing and TaqMan SNP genotyping methods involved in the testing of *M*. *leprae* drug resistance are capable of detecting other *Mycobacterium* species or nonmycobacterial species. The *Mycobacterium* species expressing the rpoB, gyrA, and folP1 genes retrieved by NCBI/gene are shown in S2 Table. In this study, no cross-detection was found for any primers/probes used for gyrA or for folP1. Positive rpoB amplicons can be distinguished as the appropriate *Mycobacterium* species using NCBI/BLAST. As the rpoB gene is also expressed in other mycobacterial species, theoretically, the method can also be performed to detect the rpoB gene in the other four *Mycobacterium* species. The results suggested no risk of interference from mycobacterial species that might be encountered when testing *M*. *leprae* for drug resistance.

Point mutations at codon 53 or 55 of the *M*. *leprae* folP1 gene have been confirmed to result in dapsone resistance [18]. folP1 mutation rates among relapsed cases have been reported to be 26% (5/19) in the Philippines (Cebu), 8.3% (2/24) in Myanmar (Yangon), 10% (1/10) in Indonesia (North Maluku and North Sulawesi) [10], 57% (8/14) in Vietnam (the central and highland regions) [17], and 9.1% (2/22) in Japan [19]. In this study, the frequencies of dapsone resistance in new and relapsed cases were 24.00% (12/50) and 26.92% (7/26), respectively. In this study, the rate of mutation of the folP1 gene in relapsed cases (26.92%, 7/26) was slightly higher than that in newly detected cases (24.00%, 12/50). These results are different from those of a previous study in Shandong, China [20], which reported frequencies of dapsone resistance in new and relapsed cases of 1.6% (1/61) and 0% (0/6), respectively. In addition, a previous study in Shandong, China, reported that the mutation rate of the rpoB gene in 52 new cases was 9.6% (5/52), though rpoB mutation was not found in new or relapsed cases in this study. These different drug resistance characteristics may be due to differences in demographics from Southwest China to North China and the small sample size of this study.

Based on the result of an antimicrobial resistance study on leprosy by the WHO surveillance network, skin biopsy specimens from MB leprosy cases at sentinel sites of 19 countries from the period 2009–2015 were included, and resistance to rifampicin, dapsone and ofloxacin according to PCR sequencing of rpoB, folP1 and gyrA gene DRDRs was studied [21]. The PCR/sequencing method has been widely applied in MB leprosy patients; however, drug resistance among PB leprosy patients and the use of FFPE specimens have seldom been discussed [15]. In this study, we developed a nested PCR and TaqMan SNP Genotyping Assay, which were used for drug resistance testing in MB and PB patients. The performance of the two methods was also assessed to detect drug resistance within DRDRs of *M*. *leprae* using DNA obtained from skin biopsy as well as from partially degraded FFPE specimens.

The three molecular methods were applied to 73 specimens with BI values ranging from 0 (no bacilli visible) to 6 (>1000 bacilli per microscopic field), thereby enabling a comparison of the efficiency of the methods between the BI≥2 and BI <2 groups (Table 4). As expected, there was a direct correlation between PCR/sequencing positivity and the BI, but surprisingly, nested PCR/seq and the TaqMan SNP Genotyping Assay increased the sensitivity dramatically, and successful detection of drug resistance was achieved with some specimens with a BI as low as 0. In addition, distinguishing heterozygous genotypes and mutant homozygous genotypes from homozygous genotypes based on an allele discrimination plot of the TaqMan SNP Genotyping Assay was easily accomplished.

Dapsone monotherapy is usually regarded as the reason for drug resistance in relapsed cases. However, only two relapsed cases with folP1 mutation had previously undergone DDS monotherapy. Of notable concern was the detection of the folP1 mutation related to drug resistance in five patients undergoing MDT who had not previously undergone DDS treatment. This may be due to irregular medication use as a result of self-treatment. Despite the known presence of dapsone resistance mutations, relapsed patients are commonly retreated with an MDT regimen that contains dapsone in Colombia [22] and China.

Our study has some limitations. It was a retrospective study with a small sample size. Thus, a prospective study with a standardized sampling method should be performed. The TaqMan SNP Genotyping Assay can detect only one SNP locus each time, and more effective detection systems, such as the TaqMan array card (TAC), should be developed. We focused on mutations only within DRDRs. Future studies should focus on additional SNPs related to drug resistance in *M*. *leprae* as well as transmission markers of leprosy.

### Conclusion

Overall, our findings highlight a new molecular approach involving nested PCR, and the TaqMan SNP Genotyping Assay offers a rapid and highly sensitive tool for testing resistance in *M*. *leprae*. In addition, the TaqMan SNP Genotyping Assay easily distinguishes heterozygous genotypes and mutant homozygous genotypes from homozygous genotypes.

Using these tools, more information on drug resistance can be obtained from skin biopsy, FFPE, and SSS specimens from leprosy patients. However, the TaqMan SNP Genotyping Assay developed in the study can only detect one SNP mutant locus at a time. Thus, new gene detection systems, such as the TAC, integrating more SNP loci of drug resistance genes into an effective assay, should be developed. Finally, additional genetic mutations related to first-line and second-line drug resistance should be considered for incorporation into future gene detection systems.

## Supporting information

S1 Table DNA concentrations (ng/μl) obtained for *Mycobacterium* species and *M*. *leprae* clinical specimens, as determined using spectrophotometry and Qubit dsDNA HS assay. (DOC)

S2 Table *Mycobacterium* species expression of the rpoB, gyrA, and folP1 genes, as reported by NCBI/gene. (DOC)
