# Supplementary material for: Nested PCR and the TaqMan SNP Genotyping Assay enhanced the sensitivity of drug resistance testing of Mycobacterium leprae using clinical specimens of leprosy patients
Source: PLoS Negl Trop Dis. 2019 Dec 27;13(12):e0007946. doi: 10.1371/journal.pntd.0007946 (PMC6934270; doi:10.1371/journal.pntd.0007946)
Supplement: S1 Table — (DOC) [file pntd.0007946.s001.doc]

S Table 1. (also S table 1 in the manuscript) DNA concentrations (ng/µl) resulting from the Mycobacterium species and M.leprae clinic specimens as determined using the [spectrophotometry](https://www.sciencedirect.com/topics/medicine-and-dentistry/spectrophotometry) and Qubit dsDNA HS Assay.

| Specimens types | Skin biopsy | | FFPE | |
| --- | --- | --- | --- | --- |
| Number of specimens | 62 | | 11 | |
| DNA concentration  Assay | [spectro-](https://www.sciencedirect.com/topics/medicine-and-dentistry/spectrophotometry)  [photometry](https://www.sciencedirect.com/topics/medicine-and-dentistry/spectrophotometry) | Qubit dsDNA HS Assay | [spectro-](https://www.sciencedirect.com/topics/medicine-and-dentistry/spectrophotometry)  [photometry](https://www.sciencedirect.com/topics/medicine-and-dentistry/spectrophotometry) | Qubit dsDNA HS Assay |
| Minimum | 9.150 | 0.1760 | 16.25 | 4.820 |
| 25% Percentile | 24.80 | 6.965 | 27.30 | 9.180 |
| Median | 49.75 | 55.70 | 101.4 | 54.40 |
| 75% Percentile | 80.85 | 81.55 | 167.0 | 102.0 |
| Maximum | 240.6 | 236.0 | 224.5 | 104.0 |
| Mean | 65.24 | 53.01 | 107.9 | 58.73 |
| Std. Deviation | 55.42 | 46.53 | 72.99 | 42.54 |
| Std. Error | 7.039 | 5.910 | 22.01 | 12.83 |
| Lower 95% CI of mean | 51.17 | 41.19 | 58.86 | 30.15 |
| Upper 95% CI of mean | 79.32 | 64.83 | 156.9 | 87.31 |
| Sum | 4045 | 3287 | 1187 | 646.1 |
