# Supplementary material for: Nested PCR and the TaqMan SNP Genotyping Assay enhanced the sensitivity of drug resistance testing of Mycobacterium leprae using clinical specimens of leprosy patients
Source: PLoS Negl Trop Dis. 2019 Dec 27;13(12):e0007946. doi: 10.1371/journal.pntd.0007946 (PMC6934270; doi:10.1371/journal.pntd.0007946)
Supplement: S2 Table — (DOC) [file pntd.0007946.s002.doc]

Stable 2. The mycobacterium species expressed RpoB, GyrA, and FolP1 gene reported by NCBI/gene.

| rpoB | gyrA | Folp1 |
| --- | --- | --- |
| Results by taxon | Results by taxon | Results by taxon |
| *Top Organisms [Tree]* | *Top Organisms [Tree]* | *Top Organisms [Tree]* |
| [*Mycobacterium tuberculosis (8)*](https://www.ncbi.nlm.nih.gov/gene/?term=rpoB+mycobacterium) | [*Mycobacterium sp. KMS (3)*](https://www.ncbi.nlm.nih.gov/gene/?term=gyrA+mycobacterium) | [*Mycobacterium tuberculosis H37Rv (1)*](https://www.ncbi.nlm.nih.gov/gene/?term=folp1+mycobacterium) |
| [*Mycobacterium leprae (2)*](https://www.ncbi.nlm.nih.gov/gene/?term=rpoB+mycobacterium) | [*Mycobacterium kansasii ATCC 12478 (3)*](https://www.ncbi.nlm.nih.gov/gene/?term=gyrA+mycobacterium) | [*Mycobacterium tuberculosis variant bovis AF2122/97 (1)*](https://www.ncbi.nlm.nih.gov/gene/?term=folp1+mycobacterium) |
| [*Mycobacterium kansasii ATCC 12478 (1)*](https://www.ncbi.nlm.nih.gov/gene/?term=rpoB+mycobacterium) | [*Mycobacterium asiaticum DSM 44297 (3)*](https://www.ncbi.nlm.nih.gov/gene/?term=gyrA+mycobacterium) |  |
| [*Mycobacterium gordonae (1)*](https://www.ncbi.nlm.nih.gov/gene/?term=rpoB+mycobacterium) | [*Mycolicibacterium fortuitum (3)*](https://www.ncbi.nlm.nih.gov/gene/?term=gyrA+mycobacterium) |  |
| [*Mycobacterium avium subsp. paratuberculosis K-10 (1)*](https://www.ncbi.nlm.nih.gov/gene/?term=rpoB+mycobacterium) | [*Mycobacterium leprae TN (2)*](https://www.ncbi.nlm.nih.gov/gene/?term=gyrA+mycobacterium) |  |
| [*Mycobacterium intracellulare ATCC 13950 (1)*](https://www.ncbi.nlm.nih.gov/gene/?term=rpoB+mycobacterium) | [*Mycobacterium avium subsp. paratuberculosis K-10 (2)*](https://www.ncbi.nlm.nih.gov/gene/?term=gyrA+mycobacterium) |  |
| [*Mycobacterium colombiense CECT 3035 (1)*](https://www.ncbi.nlm.nih.gov/gene/?term=rpoB+mycobacterium) | [*Mycobacterium intracellulare ATCC 13950 (2)*](https://www.ncbi.nlm.nih.gov/gene/?term=gyrA+mycobacterium) |  |
| [*Mycobacterium sp. KMS (1)*](https://www.ncbi.nlm.nih.gov/gene/?term=rpoB+mycobacterium) | [*Mycobacterium colombiense CECT 3035 (2)*](https://www.ncbi.nlm.nih.gov/gene/?term=gyrA+mycobacterium) |  |
| [*Mycobacterium asiaticum DSM 44297 (1)*](https://www.ncbi.nlm.nih.gov/gene/?term=rpoB+mycobacterium) | [*Mycobacterium marinum E11 (2)*](https://www.ncbi.nlm.nih.gov/gene/?term=gyrA+mycobacterium) |  |
| [*Mycobacterium interjectum (1)*](https://www.ncbi.nlm.nih.gov/gene/?term=rpoB+mycobacterium) | [*Mycobacterium tuberculosis H37Rv (2)*](https://www.ncbi.nlm.nih.gov/gene/?term=gyrA+mycobacterium) |  |
| [*Mycobacterium europaeum (1)*](https://www.ncbi.nlm.nih.gov/gene/?term=rpoB+mycobacterium) | [*Mycobacterium europaeum (2)*](https://www.ncbi.nlm.nih.gov/gene/?term=gyrA+mycobacterium) |  |
| [*Mycobacterium marinum E11 (1)*](https://www.ncbi.nlm.nih.gov/gene/?term=rpoB+mycobacterium) | [*Mycobacterium gordonae (2)*](https://www.ncbi.nlm.nih.gov/gene/?term=gyrA+mycobacterium) |  |
| [*Mycolicibacterium smegmatis MC2 155 (1)*](https://www.ncbi.nlm.nih.gov/gene/?term=rpoB+mycobacterium) | [*Mycolicibacterium smegmatis MC2 155 (2)*](https://www.ncbi.nlm.nih.gov/gene/?term=gyrA+mycobacterium) |  |
| [*Mycolicibacterium fortuitum (1)*](https://www.ncbi.nlm.nih.gov/gene/?term=rpoB+mycobacterium) | [*Mycobacteroides abscessus (2)*](https://www.ncbi.nlm.nih.gov/gene/?term=gyrA+mycobacterium) |  |
| [*Mycobacteroides abscessus (1)*](https://www.ncbi.nlm.nih.gov/gene/?term=rpoB+mycobacterium) | [*Mycobacteroides chelonae CCUG 47445 (2)*](https://www.ncbi.nlm.nih.gov/gene/?term=gyrA+mycobacterium) |  |
| [*Mycobacteroides chelonae CCUG 47445 (1)*](https://www.ncbi.nlm.nih.gov/gene/?term=rpoB+mycobacterium) | [*Mycobacterium virus Bethlehem (2)*](https://www.ncbi.nlm.nih.gov/gene/?term=gyrA+mycobacterium) |  |
| [*Corynebacterium diphtheriae (1)*](https://www.ncbi.nlm.nih.gov/gene/?term=rpoB+mycobacterium) | [*Mycobacterium phage Turj99 (2)*](https://www.ncbi.nlm.nih.gov/gene/?term=gyrA+mycobacterium) |  |
|  | [*Mycobacterium phage Violet (2)*](https://www.ncbi.nlm.nih.gov/gene/?term=gyrA+mycobacterium) |  |
|  | [*Mycobacterium tuberculosis variant bovis AF2122/97 (1)*](https://www.ncbi.nlm.nih.gov/gene/?term=gyrA+mycobacterium) |  |
|  | [*Mycobacterium interjectum (1)*](https://www.ncbi.nlm.nih.gov/gene/?term=gyrA+mycobacterium) |  |
|  | [*All other taxa (39)*](https://www.ncbi.nlm.nih.gov/gene/?term=gyrA+mycobacterium) |  |
